# Noninvasive and Safe Cell Viability Assay for Breast Cancer MCF-7 Cells Using Natural Food Pigment

**DOI:** 10.3390/biology9080227

**Published:** 2020-08-14

**Authors:** Kyohei Yamashita, Ryoma Tagawa, Yoshikazu Higami, Eiji Tokunaga

**Affiliations:** 1 Department of Physics, Faculty of Science, Tokyo University of Science, 1-3 Kagurazaka, Shinjuku-ku, Tokyo 162-8601, Japan; eiji@rs.kagu.tus.ac.jp; 2 Laboratory of Molecular Pathology and Metabolic Disease, Faculty of Pharmaceutical Sciences, Tokyo University of Science, 2641 Yamazaki, Noda, Chiba 278-8510, Japan; tagawar@rs.tus.ac.jp (R.T.); higami@rs.noda.tus.ac.jp (Y.H.)

**Keywords:** viability assay, dye exclusion test, natural food pigment, *Monascus*, human breast cancer cell, MCF-7, noninvasive, trypan blue

## Abstract

A dye exclusion test (DET) was performed to determine the viability of human breast cancer cells MCF-7, using natural food pigments as compared with trypan blue (TB), a typical synthetic dye for DET known to exhibit teratogenicity and cytotoxicity. We demonstrated that *Monascus* pigment (MP) is noninvasive to living cells and can effectively stain only dead cells. This study is the first verification of the applicability of MP to cancer cells. The appropriate MP concentration was 0.4% (0.02% as the concentration of pure MP) and all the dead cells were stained within 10 min. We found that the cell proliferation or the reduced nicotinamide adenine dinucleotide (NADH) activity of living cells was maintained over 48 h. Although 0.1% TB did not show an increase in dead cells, a marked decrease in NADH activity was confirmed. In addition, even when MP coexisted with cisplatin, staining of dead cells was maintained for 47 h, indicating stability to drugs (reagents). The cost of MP is estimated to be about 1/10 of TB. The fact that MP can be used as a cell viability determination reagent for *Euglena* and *Paramecium*, as shown in preceding papers, and also for MCF-7, as shown in this paper, indicates the possibility of application in more cells of different species.

## 1. Introduction

In vitro tests are faster, easier to perform and to quantify, usually cheaper, and allow studies of isolated steps [1]. In vitro, chemical substances such as drugs and pesticides have various cytotoxic mechanisms such as destruction of cell membranes and prevention of protein synthesis [2]. To identify cell death caused by these toxicities, the fields of toxicology and pharmacology require inexpensive, reliable, and reproducible short-term cytotoxicity and cell viability assays.

In particular, in the case of a harmless method for assessing the viability of a target cell, more detailed information can be obtained by long-term monitoring of the viability. Furthermore, since it is not necessary to prepare the same number of samples for viability assay, the cost and labor can be reduced, and also the problem of non-uniformity between samples does not occur. Therefore, there should be a potential demand for a long-term, noninvasive, safe viability assay of the same sample. 

The following conventional methods to distinguish between live and dead cells in culture have been established:

**Dye exclusion test (DET)** Cells stained with a synthetic dye such as trypan blue (TB) are judged as dead cells [3].
**Colony formation assay** The number of live cells is evaluated by the number of colonies formed on an agar culture after an inoculation of diluted cell suspension and following a definite time of culture [4].
**Enzyme activity assay** Enzymatic reaction in living cells or enzymes leaking out of dead cells are used for viability assay [5].
**Flow cytometry analysis** Dead cells are labeled with a fluorescent dye [6] and detected by fluorescence flow cytometry [7,8].
**Optical method** The dead or living state of cells is diagnosed by deflection change of the probe light beam [9].
**Electro-orientation-based assay** The positions of alternative electro-orientation of living or dead cylindrical yeast cells are dependent on the applied frequency [10].

However, these methods have difficulties such as requiring specialized techniques and equipment, damaging cells, inability to perform in situ measurement in the cultivation process, or only applicable for cells of a specific shape (non-spherical) [9,10,11]. Therefore, in order to solve these problems, we propose a DET using natural edible pigments that are safe for humans and minimally invasive to cells, as an alternative to a DET using conventional synthetic dyes that are toxic to humans and invasive to cells.

Trypan blue (TB), a vital dye, has been conventionally used for the DET. TB is a widely used diazo dye for selectively coloring dead cells or tissues. The mechanism for TB to stain dead cells is based on its negative charge which prevents the incorporation of it into the living cells with the membrane negatively charged. Therefore, living cells are not stained, but dead cells with the compromised cell membrane are stained by TB [12,13,14]. However, TB is known to cause cell health and environmental problems due to its potential teratogenic effects [15,16]. It has also been pointed out that pore formation in cell membranes is possibly induced to increase membrane permeability [13]. As other dyes for the DET, methylene blue [17,18], eosin [19], nile blue [20], and amethyst violet [21] have been used but it is known that the selective permeability of the plasma membrane is destroyed or severely impaired [3], indicating that these dyes are toxic for cells. 

In order to avoid these problems, six natural food pigments (*Monascus*, purple sweet potato, yellow gardenia, green gardenia, red beet, and *Spirulina*) and a traditional synthetic dye (TB) for viability assay are tested in this study, among which *Monascus* pigment (MP) is of particular interest. MP is extracted from the *Monascus* sp. which is a kind of filamentous fungus.

MP exhibits a red color due to a molecule of the pigment whose main component is monascorubrin [22]. It does not have a significant pH dependence of the color (although it tends to precipitate in acidic solution), has excellent stainability to proteins, and is relatively stable to heat [22]. MP is not easily affected by metal ions other than copper [23], whereas it is unstable against light irradiation, especially in an acidic condition. MP has been used for more than 1000 years as a food pigment and a folk medicine in China because an efficient production method was established using fermentation of rice. MP is a cost-effective and reproducible substrate, has variation in colors, is highly safe, and shows good solubility in water and ethanol [24,25]. Moreover, it possesses biological activities such as anticancer properties, anti-mutagenic activity, antibacterial activity, and potential anti-obesity activity [26]. Regarding the quality assurance of MP, it is based on the component standards in the Japan’s Specifications and Standards for Food Additives (JSFA) [27]. The details are described in the article of the example applied to *Paramecium* [28]. 

Animal cells for various pathological experiments do not have motility or characteristic pigments, making it difficult to judge using only visual microscopic observation. The breast cancer cell line, MCF-7 cells, which is widely available as an in vitro model for cancer research, has been used [29]. Cisplatin (cis-diamminedichloroplatinum (II)) is a widely used chemotherapeutic drug for the treatment of various types of cancer, including breast cancer [30,31]. In particular, MCF-7 cells are widely used in studies of estrogen receptor (ER, positive breast cancer cells [32,33].

To verify that this method is applicable to a wide variety of cells, first, we examined whether natural pigments are not toxic to human breast cancer cells MCF-7 cells in the presence or absence of cisplatin and if they can be replaced with conventional synthetic dyes for viability assay. Then, the effect of natural pigments on cells with different properties was investigated, and the results were compared between the unicellular green alga *Euglena* [34] and the protozoan *Paramecium* [28], which differ in cell structure. 

## 2. Materials and Methods

### 2.1. Sample Preparation

Human breast cancer MCF-7 cells were cultured stationarily in Eagle’s Minimal Essential Medium (E-MEM) (Wako, Osaka, Japan) supplemented with 10% FBS (Thermo Fisher Scientific, Waltham, MA, USA), 1% penicillin streptomycin (P/S) (Merck KGaA, Darmstadt, Germany), 10 μg/mL insulin (Wako), 1% MEM nonessential amino acids solution, and 1 mM sodium pyruvate solution at 37 °C and 5% CO_2_ on a 6-well plate (92006, TPP), 12-well plate (92012, TPP), and 96-well plate (92096, TPP). Cisplatin was dissolved in 90% dimethyl sulfoxide in phosphate buffered saline (90% DMSO in PBS) before use. Incubation period before and after addition of pigment for each experiment is shown in Table 1.

### 2.2. Bright-Field Observation of Cells

**Figure 1:**
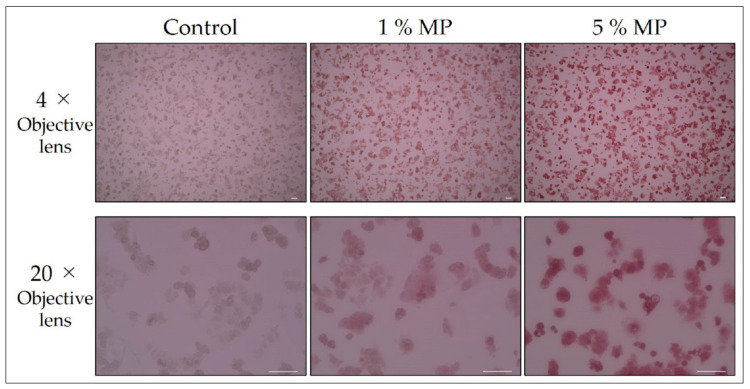
Magnification of observation and staining of dead cells (microwave treatment). All scale bars designate 100 μm.

**Figure 2:**
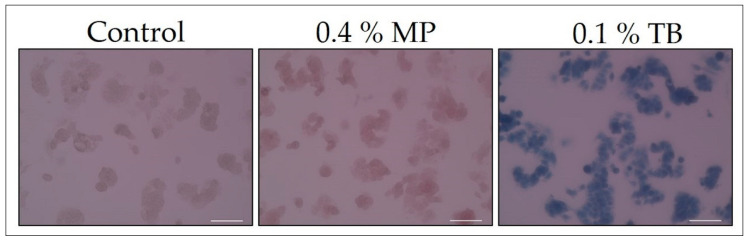
Confirmation of staining of dead cells (microwave treatment). All scale bars designate 100 μm.

**Figure 3:**
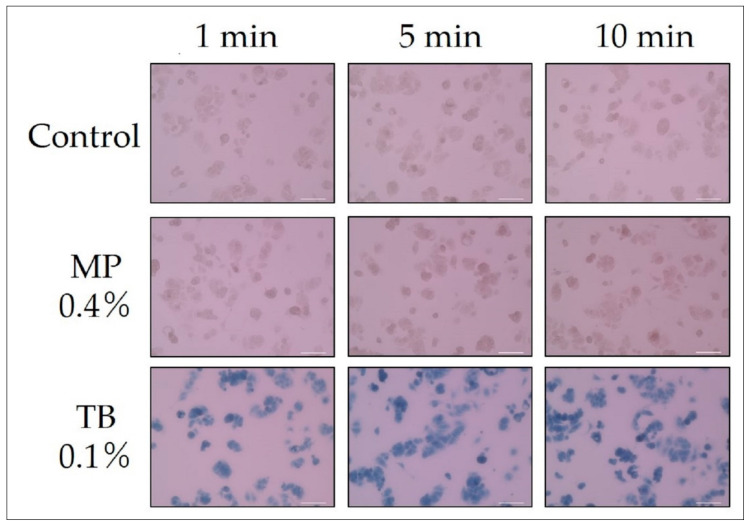
Immersion time and staining of dead cells (microwave treatment). All scale bars designate 100 μm.

**Figure 4:**
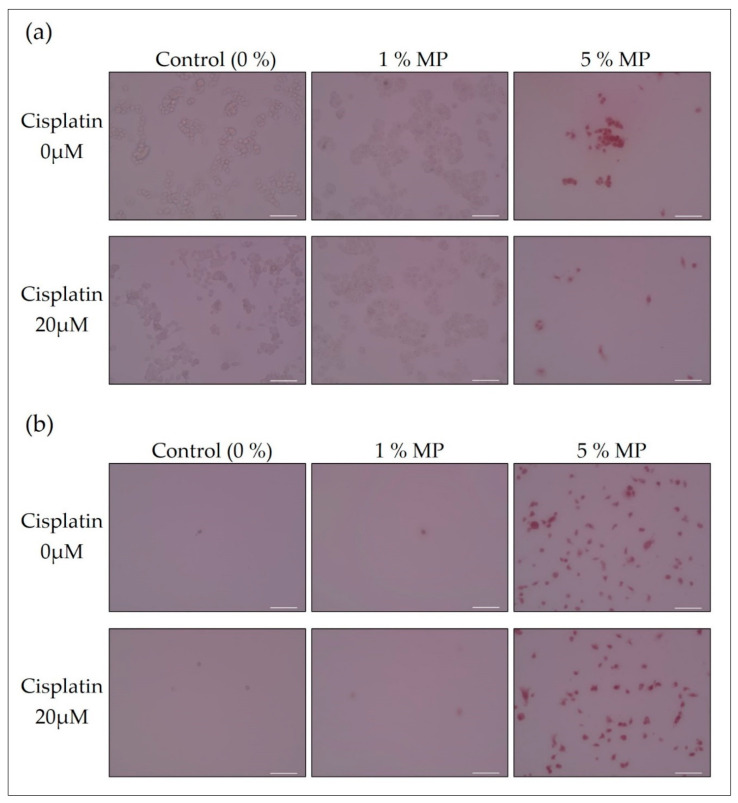
Staining of cells in a mixed solution of MP and cisplatin. (**a**) Cells on the bottom of dishes; (**b**) Floating cells. All scale bars designate 100 μm.

**Figure 5:**
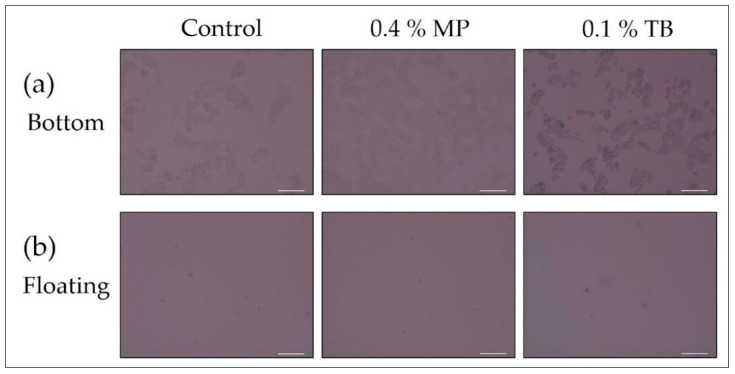
Confirmation of noninvasiveness of pigment (47 h pigment immersion). (**a**) Cells on the bottom of dishes; (**b**) Floating cells. All scale bars designate 100 μm.

The following were used for bright-field observation in each test (listed in Table 2):

- A fluorescence microscope, BZ-9000;
- A 4× objective lens, NA 0.20, CFI Plan Apochromat Lambda 4X, Nikon;
- A 20× objective lens, NA 0.75, CFI Plan Apochromat Lambda 20X, Nikon;
- Exposure time, 1/900 sec (4× objective lens, Figure 1);
- Exposure time, 1/130 sec (20× objective lens, Figure 2);
- Exposure time, 1/80 sec (20× objective lens, Figure 3);
- Exposure time, 1/130 sec (20× objective lens, Figure 4 and Figure 5).

Only the experiments in Table 3. “Pigment usable/unusable for viability assay” were performed under the following conditions (the objective lens is the same as above):

- 4× objective lens, 1/800 sec or 1/900 sec.

### 2.3. Preparation of Dead Cells of MCF-7 Cells (Common for All Experiments)

Dead cells were obtained by the following two treatments:

**Microwave (MW) treatment** Cells were treated with microwave at 2.45 GHz until it was boiled.
**Cisplatin treatment** Cisplatin (Wako) was added to the culture and adjusted to a final concentration of 20 μM. According to the report, the survival rate of MCF-7 cells at 20 μM of cisplatin was estimated to be about 80% [35]. In this report, the relationship between survival rate and cisplatin concentration was 85.67% (12 μM) and 76.03% (25 μM) (Figure 1B in reference [35], not in this paper).

### 2.4. Pigment for Viability Assay

The following two types of pigments were used in this study for viability assay (the pigment concentrations in each experiment are shown in Table 2):

**Natural food pigment** Food pigment Red (*Monascus*, Watashinodaidokoro Co., Ltd. http://watashinodaidokoro.net/);
**Synthetic dye** Trypan blue (Merck KGaA).

Here, the component of “Food pigment Red” contains 95% *w*/*w* dextrin by weight, and the remaining 5% is pure MP. However, in the preceding papers that used *Euglena* and *Paramecium* as samples [28,34], the concentration of “Food pigment Red” was described as the concentration of “MP”. (Cf. The concentration of MP shown in Table 2 is the concentration of “Food pigment Red” and the concentration of “pure” MP is 5% of that value.).

### 2.5. Confirmation of Staining by Natural Pigment

The MCF-7 cells were plated at a density of 5.0 × 10^5^ cells/well in 6-well plates (10 mm^2^/well). After 2 days, the MCF-7 cells were treated with six natural food pigments (*Monascus*, 0.5% *W*/*W* (purple sweet potato) or 1% *W*/*W* (*Monascus*, yellow gardenia, green gardenia, red beet, or *Spirulina*) in the presence or absence of 20 μM cisplatin, for 12 h. Then, cells mixed with *Monascus* or green gardenia were washed with PBS. Samples mixed with other pigments were not washed with PBS. Cells were observed with a bright field microscope (4× objective lens). The pigment that enabled easy visual discrimination of stained cells was defined as usable as a reagent for viability assay. The results are shown in Table 3.

### 2.6. Magnification of Observation and Staining of Dead Cells

The MCF-7 cells were plated at a density of 3.8 × 10^5^ cells/well in 6-well plates. After 2 days, the MCF-7 cells were microwaved until the medium boiled. After discarding the medium, cells were treated with 1 or 5% MP for 20 min, and then washed three times with PBS at room temperature. Cells treated with no pigment were used as the “control”. Cells on the bottom of the dish were observed with a bright-field microscope (4× or 20× objective lens). The results are shown in Figure 1.

### 2.7. Confirmation of Staining of Dead Cells

The MCF-7 cells were plated at a density of 1.5 × 10^5^ cells/well in 12-well plates (4 mm^2^/well). After 2 days, the MCF-7 cells were microwaved until the medium boiled. After discarding the medium, cells were treated with 0.4% MP or 0.1% TB for 20 min, and then washed three times with PBS at room temperature. Cells treated with no pigment were used as the “control”. Cells on the bottom of the dish were observed with a bright-field microscope (20× objective lens). The results are shown in Figure 2.

### 2.8. Immersion Time and Staining of Dead Cells

The MCF-7 cells were plated at a density of 1.5 × 10^5^ cells/well in 12-well plates (4 mm^2^/well). After 2 days, the MCF-7 cells were microwaved until the medium was boiled. After discarding the medium, cells were treated with 0.4% MP or 0.1% TB for 20 min, and then washed three times with PBS at room temperature. Cells treated with no pigment were used as the “control”. Cells on the bottom of the dish were observed with a bright-field microscope (20× objective lens). The results are shown in Figure 3.

### 2.9. Staining of Cells in A Mixed Solution of MP and Cisplatin

The MCF-7 cells were plated at a density of 3.8 × 10^5^ cells/well in 6-well plates (10 mm^2^/well). After 2 days, the MCF-7 cells were treated with 1 or 5% MP in the presence or absence of 20 μM cisplatin for 14 h. The medium was collected and the cells on the bottom of 6-well plates were washed three times with PBS (“bottom” sample). The medium was centrifuged at 200× *g* for 5 min, at 4 °C. After discarding the supernatant, the pellet was washed by PBS and centrifuged again. The pellet was suspended by PBS and moved to new 12-well plates as floating cell sample (“floating” sample). Cells treated with no pigment were used as the “control”. Cells were observed with a bright-field microscope (20× objective lens). The results are shown in Figure 4.

### 2.10. Confirmation of Noninvasiveness of Pigment

The MCF-7 cells were plated at a density of 1.5 × 10^5^ cells/well in 12-well plates (4 mm^2^/well). After 2 days, the MCF-7 cells were treated with 0.4% MP or 0.1% TB for 47 h. The medium was collected and the cells on the bottom of the 6-well plates were washed three times by PBS (bottom sample). The medium was centrifuged at 200× *g* for 5 min, at 4 °C. After discarding the supernatant, the pellet was washed by PBS and centrifuged again. The pellet was suspended by PBS and moving new 12-well plates as floating cell sample (floating sample). Cells treated with no pigment were used as the “control”. Cells were observed with a bright-field microscope (20× objective lens). The results are shown in Figure 5.

### 2.11. Cytotoxicity Assay (WST-8)

**Figure 6:**
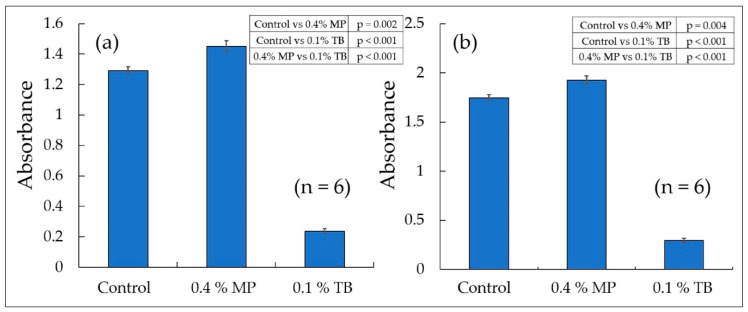
Cytotoxicity assay (WST-8). (**a**) 24 h; (**b**) 48 h.

Cell Counting Kit-8 (Dojindo Molecular Technologies, Inc.) is a cell proliferation/cytotoxicity measurement kit that uses the water-soluble tetrazolium salt WST-8 (2-(2-methoxy-4-nitrophenyl)-3-(4-nitrophenyl)-5-(2,4-disulfophenyl)-2H-tetrazolium) as a coloring reagent. When WST-8 is reduced, WST-8 produces orange, water-soluble formazan with a maximum absorption around 460 nm. WST-8 is reduced by reduced nicotinamide adenine dinucleotide (NADH) produced by dehydrogenase in the cell via 1-methoxy PMS [36]. Since the absorbance is proportional to the number of cells and the enzymatic activity of the cells, it serves as an indicator of the health of the cells. The MCF-7 cells were plated at a density of 1.3 × 10^4^ cells/well in 96-well plates (0.35 mm^2^/well). After 2 days, the MCF-7 cells were treated with 100 μL of 0.4% MP or 0.1% TB for 24 or 48 h. Then, 10 μL of WST-8 was added. Samples were incubated for 3 h. Cells treated with no pigment were used as the “control”. Samples were measured for absorbance (TECAN Nano Quant Infinite M200 Pro, Tecan, Zurich, Switzerland). From the sample absorbance (450 nm), the absorbance of a solution in which an equivalent amount of the pigment (Figure 6) and WST-8 were added to the fresh growth medium as a background was subtracted. Samples were measured in six independent wells. Statistical analysis was performed by one-way analysis of variance (ANOVA) and Tukey test using BellCurve for Excel software (Social Survey Research Information Co., Ltd., Tokyo, Japan). The average value of “the absorbance ± standard error (*n* = 6)” is shown in Figure 6 and the results of the Tukey test are shown in Figure 6. A *p*-value <0.05 was considered to be significant.

## 3. Results

### 3.1. Confirmation of Staining by Natural Pigment

Six natural food pigments (*Monascus*, purple sweet potato, yellow gardenia, green gardenia, red beet, and *Spirulina*) were added to the cell suspension (1%). For each sample with pigment added, a sample with cisplatin added (20 μM) and a control sample with no pigment added were prepared. The pigments that could be easily visually discriminated by bright field microscope observation after 12 h, and in which more cells were stained in the cisplatin-added sample than in the control sample, were made usable as a reagent for viability assay. Trypan blue was determined to be usable because we confirmed that it was a conventional reagent for viability assay and that it could be usable by other tests (Figure 2, Figure 3 and Figure 5). The data are shown in Supplementary Material, and the results are summarized in Table 3.

As a result of DET with six types of natural pigments, it was found that only MP could be used as a reagent for viability assay. A characteristic of MP that is different from other natural pigments is strong staining of intracellular proteins. The mechanism of staining with TB also results from binding to intracellular proteins [14]. In the preceding papers on DET of *Euglena* and *Paramecium*, long-term viability could be determined with anthocyanin pigments (purple sweet potato and red cabbage dye) in addition to MP [28,34]. This was thought to be due to differences in cell structure. Therefore, it was suggested that high affinity for the intracellular protein is a more suitable dye condition for DET.

### 3.2. Magnification of Observation and Staining of Dead Cells

Cell death of MCF-7 cells was induced by microwave exposure. After discarding the medium, dead cells were treated with MP (1 or 5%) for 20 min, and then washed with PBS. Cells treated with no pigment were used as the “control”. Cells on the bottom of the dish were observed with a bright-field microscope (4× or 20× objective lens).

From Figure 1, one can visually confirm the staining of the MW treated dead cell when observing the 1% MP with a 4× objective lens. In the observation of 5% MP dead cells with a 4× objective lens, even deeper staining was obtained, and it was found that in this concentration range, dead cells were deeply stained in proportion to the MP concentration. In addition, observation with a 20× objective lens enabled us to distinguish individual stained cells with higher visibility. However, with respect to living cells, at a concentration of 0.6%, although the morphology of the cells did not change in 24 h, the results showed that the cells deformed into spheres in 48 h (data not shown). By contrast, no morphological change was observed at a concentration of 0.4%. Therefore, in the case where MP is used instead of TB in conventional DET without performing long-term monitoring of viability assay, it is advantageous to use high concentration for the purpose of enhancing visibility. Nevertheless, when performing long-term monitoring of viability assay, it is necessary to adjust the MP concentration to 0.4%.

### 3.3. Confirmation of Staining of Dead Cells

Cell death of MCF-7 cells was induced by microwave exposure. After discarding the medium, dead cells were treated with 0.4% MP or 0.1% TB for 20 min, and then washed with PBS. Cells treated with no pigment were used as the “control”. Cells on the bottom of the dish were observed with a bright-field microscope (20× objective lens). Because of the light red background, it looks as if the cells are not clearly stained, however, staining of dead cells was sufficiently confirmed by visual observation with a microscope.

Figure 2 shows the result of confirming the staining of MW treated dead cells by 0.4% MP, and staining was clearly confirmed against the “control” containing no pigment. Clear staining was confirmed also with 0.1% TB.

### 3.4. Immersion Time and Staining of Dead Cells

Cell death of MCF-7 cells was induced by microwave exposure. After discarding the medium, dead cells were treated with MP (1 or 5%) for the indicated times, and then washed with PBS. Cells treated with no pigment were used as the “control”. Cells on the bottom of the dish were observed with a bright-field microscope (20× objective lens). Because of the light red background, it looks as if the cells are not clearly stained, however, staining of dead cells was sufficiently confirmed by visual observation with a microscope.

Figure 3 shows the state of staining of MW treated dead cells at each elapsed time from the addition of the pigment to the cell suspension. On the one hand, in the 0.4% MP, almost all cells were stained in 5 min, and staining of all cells was confirmed in 10 min. The time until this dyeing is within a practical range. On the other hand, almost all cells of the TB (0.1%) were stained in 1 min. There is no rigorous comparison of visibility, but TB is at a higher concentration, considering that the “pure MP” concentration is 5% of the amount of MP added to the medium (i.e., pure MP concentration is 0.02%). Therefore, it is reasonable to judge that the higher pigment concentration in TB resulted in a shorter staining time.

### 3.5. Staining of Cells in a Mixed Solution of MP and Cisplatin

The MCF-7 cells were treated with MP (1 or 5%) in the presence or absence of cisplatin for 14 h. (a) Cells on the bottom of the dish and (b) floating cells in the medium were washed with PBS. Cells treated with no pigment were used as the “control”. Cells were observed with a bright-field microscope (20× objective lens).

MP stained dead cells when 20 μM cisplatin was present in the live cell suspension, as well as when it was not (Figure 4). Living MCF-7 attached to the bottom of the dish, whereas dead cells tended to float away from it. The detachment of dead cells in MCF-7 could be due to the weakening of cell adhesion due to cleavage of β-catenin because β-catenin forms a complex with cadherin involved in cell adhesion [37]. Regardless of the presence or absence of cisplatin, the “control” containing no pigment and the sample with 1% MP showed very few floating cells, and 5% MP showed many cells. Regarding cell staining, at 24 h and 48 h, 1% MP stained only floating cells, and 5% MP stained both cells on the dish and floating cells, regardless of the presence or absence of cisplatin. Therefore, it was confirmed that MP was not affected by the presence of cisplatin for 48 h and stably functions as a reagent of viability assay. This is thought to be due to the fact that MP is less affected by metal ions other than copper [23], and the platinum complex cisplatin did not affect staining. High concentrations of MP were toxic to MCF-7, as almost all cells were stained with 5% MP, in the presence or absence of cisplatin. From the previous report [38], the cell proliferation of MCF-7 at MP concentrations of 0.001%, 0.01%, and 0.1% were 100%, 140%, and 0%, respectively. The cell proliferation values shown here are read from the graphs in the report. When 1% and 5% of the MP concentration in Figure 4 are converted into “pure MP concentration”, they are 0.05% and 0.25%, respectively. Therefore, the tendency about the MP concentration and the health condition of the cell coincide.

### 3.6. Confirmation of Noninvasiveness of Pigment

The MCF-7 cells were treated with 0.4% MP or 0.1% TB for 47 h. (a) Cells on the bottom of the dish and (b) floating cells from the medium were washed with PBS. Cells treated with no pigment were used as the “control”. Cells were observed with a bright-field microscope (20× objective lens). Due to the light red background, it looks as if the cells are not clearly stained, however, staining of dead cells was sufficiently confirmed by visual observation with a microscope.

Figure 5 shows a bright-field microscopic image of the living cells after 47 h at the MP concentration (0.4%) at which the viability assay can be performed. Almost all cells attached to the bottom of the dish were not stained by 0.4% MP. This was the same for 0.1% TB. The number of cells at 0.4% MP was increased against the “control” containing no pigment, while that at 0.1% TB was comparable to that of the “control”. The number of floating cells was almost the same as that of the “control” for both pigments, and the number was small. In addition, the staining of floating cells is not so clear for both pigments as compared with the cells attached to the bottom of the dish. In particular, the 0.4% MP floating cells did not appear to be stained, but this was due to the defocus, and it was confirmed that they could be visually discriminated.

### 3.7. Immersion Time and Staining of Dead Cells

The MCF-7 cells were treated with 0.4% MP or 0.1% TB for (a) 24 h or (b) 48 h. Then, the WST-8 assay was performed. Cells treated with no pigment were used as the “control”. Samples were measured for absorbance (450 nm) and the average value of “the absorbance ± standard error (*n* = 6)” was calculated. Differences between values were analyzed using a Tukey test (* *p* < 0.05 and ** *p* < 0.01). The results of the Tukey test are shown in Figure 6. Although no significant decrease in cell number was observed at 0.1% TB, the absorbance shows significantly lower values than the “control” and 0.4% MP.

Since 0.4% MP was the upper limit concentration without cell morphological change, the toxicity test was conducted only at this concentration. The WST-8 (2-(2-methoxy-4-nitrophenyl)-3-(4-nitrophenyl)-5-(2,4-disulfophenyl)-2H-tetrazolium) assay was performed at 24 h and 48 h after pigment addition to examine the health of live cells in the dye solution (Figure 6). At both times, the absorbance of 0.1% TB was significantly lower than the other samples, suggesting a decrease in cell number or a decrease in enzyme activity (Figure 6). Microscopic observation showed that the number of cells in 0.1% TB was comparable to that of the other samples (control, 0.4% MP), and that almost no cells were stained, as shown in Figure 5. Therefore, on the one hand, the enzymatic activity is considered to be remarkably suppressed in the living cells of 0.1% TB. On the other hand, 0.4% MP is noninvasive to cells due to its higher absorbance than the “control”.

## 4. Discussion 

As a result of DET using six types of natural pigments, only MP stained dead cells of MCF-7 with high visibility. A unique property of MP is that it has a high affinity for intracellular proteins. Since MP can be applied to DET even in *Euglena* and *Paramecium*, the abovementioned properties of MP were expected to be versatile in that they could target more cells of different species. The MP concentrations shown below are that of the commercial products and the concentration of pure MP is 5% of that. By adjusting the MP concentration to 0.4% (pure MP concentration 0.02%), all the MW treated dead cells were stained within 10 min after the addition of the pigment, and long-term viability assay over 48 h was realized. At this concentration there is no morphological change of the cells and no inhibition of cell growth or reduction of NADH, so it is noninvasive. Dead cells became more intense and the visibility increased in proportion to the concentration of MP. In fact, almost all cells were stained at 1% (Figure 1 and Figure 4). In this case, long-term noninvasive DET could be difficult because the morphological change occurred on the second day at 0.6%, but it would have the advantages of higher safety and less environmental impact than conventional DET with TB. Furthermore, when the MP concentration is 0.4% and the TB concentration is 0.1%, the cost of MP is estimated to be about 1/10 of TB, which is economical. As a similar method, a technique with erythrosine B (EB, also known as Red No. 3) used as a food additive was developed [39]. This synthetic colorant is an edible tar dye that does not pass through biological membranes and is compatible with automatic cell counters. Because EB was suspected of being carcinogenic, the FDA (Food and Drug Administration) banned the use in food (1990) [40,41]. Although the ban on EB was removed afterwards, most of the EB in the USA has been replaced with Allura Red (also known as Red 40). However, Allura Red is banned in many European countries because it is an azo dye [42,43]. Therefore, it is difficult to monitor the noninvasive viability assay of a synthetic food colorant over a long period of time because of the concern regarding the influence on the human body. At a concentration of TB of 0.1%, no increase in dead cells was observed, but NADH activity was significantly suppressed, indicating that a heavy load was imposed on the cells. Therefore, MP was confirmed to be less invasive and less costly than TB. In addition, even when a metal complex anticancer agent such as cisplatin coexists, stable staining of dead cells is maintained. For example, a further practical use of this technique is expected in cooperation with a technique for distinguishing normal cells from cancer cells based on the difference in staining properties of edible natural or synthetic dyes under a multiphoton laser microscope [44]. 

## 5. Conclusions

As a result of DET using six kinds of natural pigments, only MP with high affinity for proteins stably stained dead cells of MCF-7 with cisplatin. This property of MP is expected to be versatile in that they can target more cells of various species. Dead cells stained more strongly in proportion to the concentration of MP, and visibility was increased, but morphological changes of cells were observed. In this case, long-term non-invasive DET may be difficult, but it has the advantage of being more safely, less environmentally impactive, and more economical than traditional DET using TB. Therefore, if synthetic dyes for DET are replaced with a natural food pigment, the burden on the cells in the viability assay in basic research such as drug discovery will be reduced, and it will be used as an additive to the culture solution. These advantages are not seen with synthetic dyes.

## 6. Patents

Title, “Method and Kit for Cell Viability Assay”; patent application number, 2018-241789; date, 25 December 2018.

## Figures and Tables

**Table 1:** Incubation period before and after addition of pigment.

Experimental Item	Table/Figure Number	Incubation Period (Before Pigment Addition)	Incubation Period (After Pigment Addition)
Confirmation of staining by natural pigment	Table S3, Supplementary Material	2 day	12 h
Magnification of observation and staining of dead cells	Figure 1	2 day	20 min
Confirmation of staining of dead cells	Figure 2	2 day	20 min
Immersion time and staining of dead cells	Figure 3	2 day	1 min, 5 min, 10 min
Staining of cells in a mixed solution of MP and cisplatin	Figure 4	2 day	14 h
Confirmation of noninvasiveness of pigment	Figure 5	2 day	47 h
Cytotoxicity assay (WST-8 *), Tukey test	Figure 6	2 day	24 h, 48 h

* 2-(2-methoxy-4-nitrophenyl)-3-(4-nitrophenyl)-5-(2,4-disulfophenyl)-2H-tetrazolium.

**Table 2:** Concentration of added reagent and observation magnification in each examination.

Experimental Item	Table/Figure Number	Pigment/Dye Concentration (%)			Cisplatin (μM)	Objective Lens
		Control	MP	TB		
Confirmation of staining by natural pigment	Table 3	-	1 ^※^	-	0	4×/20×
Magnification of observation and staining of dead cells	Figure 1	0	1, 5	-	0	4×/20×
Confirmation of staining of dead cells	Figure 2	0	0.4	0.1	0	20×
Immersion time and staining of dead cells	Figure 3	0	0.4	0.1	0	20×
Staining of cells in a mixed solution of MP and cisplatin	Figure 4	0	1, 5	-	20	20×
Confirmation of noninvasiveness of pigment	Figure 5	0	0.4	0.1	0	20×
Cytotoxicity assay (WST-8)	Figure 6	0	0.4	0.1	0	-

^※^ The pure MP concentration is 5% of the value in the table (cf. 0.4% MP in the table means 0.02% “pure MP” concentration).

**Table 3:** Pigment usable/unusable for viability assay.

Natural Food Pigment						Synthetic Dye
*Monascus*	Purple sweet potato	Yellow gardenia	Green gardenia	Red beet	*Spirulina*	Trypan blue
◯	×	×	×	×	×	◯

◯, usable as a reagent for viability assay; ×, unusable as a reagent for viability assay.

## Supplementary Materials

The following are available online at https://www.mdpi.com/2079-7737/9/8/227/s1, Figure S1: Confirmation of staining of dead cells, Figure S2: Confirmation of staining of dead cells.

## References

[1] Moleiro A.F., Conceição G., Leite-Moreira A.F., Rocha-Sousa A. (2017). A Critical Analysis of the Available In Vitro and Ex Vivo Methods to Study Retinal Angiogenesis. J. Ophthalmol..

[2] Aslantürk Ö.S. (2017). In Vitro Cytotoxicity and Cell Viability Assays: Principles, Advantages, and Disadvantages. Genotoxicity—A Predictable Risk to Our Actual World.

[3] Bonora A., Mares D. (1982). A simple colorimetric method for detecting cell viability in cultures of eukaryotic microorganisms. Curr. Microbiol..

[4] Collins C.H., Christopher H., Lyne P.M. (1985). Microbiological Methods.

[5] Kaja S., Payne A.J., Naumchuk Y., Koulen P. (2017). Quantification of lactate dehydrogenase for cell viability testing using cell lines and primary cultured astrocytes. Curr. Protoc. Toxicol..

[6] Hamalainen-Laanaya H.K., Orloff M.S. (2012). Analysis of cell viability using time-dependent increase in fluorescence intensity. Anal. Biochem..

[7] Yamada K., Suzuki H., Takeuchi T., Kazama Y., Mitra S., Abe T., Goda K., Suzuki K., Iwata O. (2016). Efficient selective breeding of live oil-rich *Euglena gracilis* with fluorescence-activated cell sorting. Sci. Rep..

[8] Iwata O., Yamada K., Itou T., Ozeki Y., Suzuki K., Goda K. (2017). Technology for Developing Super Microalgal Biofuels. Seibutsu Butsuri.

[9] Wu X.Z., Terada S. (2005). Noninvasive diagnosis of a single cell with a probe beam. Biotechnol. Prog..

[10] Suga M., Kunimoto A., Shinohara H. (2017). Non-invasive, electro-orientation-based viability assay using optically transparent electrodes for individual fission yeast cells. Biosens. Bioelectron..

[11] (2013). Cells Detected, Alive or Dead. https://www.biocompare.com/Editorial-Articles/131962-Cells-Detected-Alive-or-Dead/.

[12] Trypan Blue—An overview Science Direct Topics. https://www.sciencedirect.com/topics/biochemistry-genetics-and-molecular-biology/trypan-blue.

[13] Tran S.L., Puhar A., Ngo-Camus M., Ramarao N. (2011). Trypan Blue Dye Enters Viable Cells Incubated with the Pore-Forming Toxin HlyII of Bacillus cereus. PLoS ONE.

[14] Strober W. (2015). Trypan Blue Exclusion Test of Cell Viability. Curr. Protoc. Immunol..

[15] Beck F., Lloyd J.B. (1964). Dosage—Response Curves for the Teratogenic Activity of Trypan Blue. Nature.

[16] Tsaousis K.T., Kopsachilis N., Tsinopoulos I.T., Dimitrakos S.A., Kruse F.E., Welge-Luessen U. (2013). Time-dependent morphological alterations and viability of cultured human trabecular cells after exposure to Trypan blue. Clin. Exp. Ophthalmol..

[17] Tampion J., Tampion M.D. (1987). Immobilized Cells: Principles and Applications.

[18] Feizi A., Zhang Y., Greenbaum A., Guziak A., Luong M., Lok Chan R.Y., Berg B., Ozkan H., Luo W., Wu M. (2016). Rapid, portable and cost-effective yeast cell viability and concentration analysis using lensfree on-chip microscopy and machine learning. Lab Chip.

[19] Schrek R. (1936). A Method for Counting the Viable Cells in Normal and in Malignant Cell Suspensions. Am. J. Cancer.

[20] Scharff T.G., Maupin W.C. (1960). Correlation of the metabolic effects of benzalkonium chloride with its membrane effects in yeast. Biochem. Pharmacol..

[21] Novelli A. (1962). Amethyst violet as a stain for distinguishing cells with a damaged membrane from normal cells. Experientia.

[22] Shimizu T., Nakamura M., Fuji M. (2001). Edible Natural Pigment (New edition).

[23] Kiriya Chemi: Monascus Colour. http://www.kiriya-chem.co.jp/tennen/koji_red.html.

[24] Chen W., Chen R., Liu Q., He Y., He K., Ding X., Kang L., Guo X., Xie N., Zhou Y. (2017). Orange, red, yellow: Biosynthesis of azaphilone pigments in Monascus fungi. Chem Sci.

[25] Wang T., Lin T. (2007). Monascus Rice Products. Advances in Food and Nutrition Research.

[26] Feng Y., Shao Y., Chen F. (2012). Monascus pigments. Appl. Microbiol. Biotechnol..

[27] Japan’s Specifications and Standards for Food Additives (D. Monographs). http://dfa-j/shokuten_kikaku_j.html.

[28] Yamashita K., Tokunaga E. (2020). Noninvasive and safe cell viability assay for Paramecium using natural pigment extracted from food. Sci. Rep..

[29] Comşa Ş., Cîmpean A.M., Raica M. (2015). The Story of MCF-7 Breast Cancer Cell Line: 40 years of Experience in Research. Anticancer. Res..

[30] Martinho N., Santos T.C.B., Florindo H.F., Silva L.C. (2019). Cisplatin-Membrane Interactions and Their Influence on Platinum Complexes Activity and Toxicity. Front. Physiol..

[31] Prabhakaran P., Hassiotou F., Blancafort P., Filgueira L. (2013). Cisplatin Induces Differentiation of Breast Cancer Cells. Front. Oncol..

[32] Sweeney E.E., McDaniel R.E., Maximov P.Y., Fan P., Jordan V.C. (2012). Models and mechanisms of acquired antihormone resistance in breast cancer: Significant clinical progress despite limitations. Horm. Mol. Biol. Clin. Investig..

[33] Gianfredi V., Nucci D., Vannini S., Villarini M., Moretti M. (2017). In vitro Biological Effects of Sulforaphane (SFN), Epigallocatechin-3-gallate (EGCG), and Curcumin on Breast Cancer Cells: A Systematic Review of the Literature. Nutr. Cancer.

[34] Yamashita K., Yamada K., Suzuki K., Tokunaga E. (2019). Noninvasive and safe cell viability assay for Euglena gracilis using natural food pigment. PeerJ.

[35] Al-Taweel N., Varghese E., Florea A.M., Büsselberg D. (2014). Cisplatin (CDDP) triggers cell death of MCF-7 cells following disruption of intracellular calcium ([Ca^2+^]_i_) homeostasis. J. Toxicol. Sci..

[36] Cell Counting Kit-8 (Technical Manual). https://www.dojindo.co.jp/manual/CK04e.pdf.

[37] Steinhusen U., Badock V., Bauer A., Behrens J., Wittman-Liebold B., Dörken B., Bommert K. (2000). Apoptosis-induced Cleavage of β-Catenin by Caspase-3 Results in Proteolytic Fragments with Reduced Transactivation Potential. J. Biol. Chem..

[38] Okubo T., Kano I. (2003). Studies on estrogenic activities of food additives with human breast cancer MCF-7 cells and mechanism of estrogenicity by BHA and OPP. Yakugaku Zasshi J. Pharm. Soc. Jpn..

[39] Kim S.I., Kim H.J., Lee H.J., Lee K., Hong D., Lim H., Cho K., Jung N., Yi Y.W. (2016). Application of a non-hazardous vital dye for cell counting with automated cell counters. Anal. Biochem..

[40] Jennings A.S., Schwartz S.L., Balter N.J., Gardner D., Witorsch R.J. (1990). Effects of oral erythrosine (2′,4′,5′,7′-tetraiodofluorescein) on the pituitary-thyroid axis in rats. Toxicol. Appl. Pharmacol..

[41] Ap F.D.A. (1990). Limits Red Dye No. 3. The New York Times.

[42] Food Dyes: Harmless or Harmful?. https://www.healthline.com/nutrition/food-dyes.

[43] PubChem Erythrosin B (COMPOUND SUMMARY). https://pubchem.ncbi.nlm.nih.gov/compound/3259.

[44] Mizoguchi A., Fujiwara T., Tanaka K., Wang S., Sai K., Kimura K. (2014). Vital Stain. European Patent.

