# Supplementary material for: Noninvasive and Safe Cell Viability Assay for Breast Cancer MCF-7 Cells Using Natural Food Pigment
Source: Biology (Basel). 2020 Aug 14;9(8):227. doi: 10.3390/biology9080227 (PMC7463555; doi:10.3390/biology9080227)
Supplement: Supplementary file 1 [file biology-09-00227-s001.pdf]

biology

Supplementary material

Noninvasive and safe cell viability assay for breast cancer MCF-7 cells using natural food pigment

Kyohei Yamashita [1*], Ryoma Tagawa [2], Yoshikazu Higami [2], and Eiji Tokunaga [1]

[1] Department of Physics, Faculty of Science, Tokyo University of Science, 1-3 Kagurazaka, Shinjuku-ku, Tokyo 162-8601, Japan
[2] Laboratory of Molecular Pathology and Metabolic Disease, Faculty of Pharmaceutical Sciences, Tokyo University of Science, 2641 Yamazaki, Noda, Chiba 278-8510, Japan
* Correspondence: yamashita.kyh@hotmail.com; Tel.: +81-3- 5228-8214

Received: 01.04.2020; Accepted: date; Published: date

Supplementary material

In this study, we conducted experiments using *Monascus* pigment as a reagent of viability assay and verified its usefulness. Prior to that, pre-tests were conducted on six natural pigments (*Monascus*, Purple sweet potato, Yellow gardenia, Green gardenia, Red beet, Spirulina). The following is the raw data of Table 3 in the text.

MCF-7 cells were plated at a density of 5.0×10^5 cells/well in 6 well-plates (10 mm^2/well). After 2 days, MCF-7 cells were treated with six natural food pigments (0.5% W/W (Purple sweet potato) or 1% W/W (*Monascus*, Yellow gardenia, Green gardenia, Red beet, Spirulina) in the presence or absence of 20 μM cisplatin for 12 h. Then, cells mixed with *Monascus* or Green gardenia were washed with PBS. Samples mixed with other pigments were not washed with PBS, because with these pigments, no clear shading of the added pigments was observed in all cells, and it was judged to be difficult to distinguish between live cells and dead cells. Cells were observed with a bright field microscope (4 × objective lens). The pigment that enables easy visual discrimination of stained cells was defined as usable as a reagent for viability assay. The results are shown in Table 3.

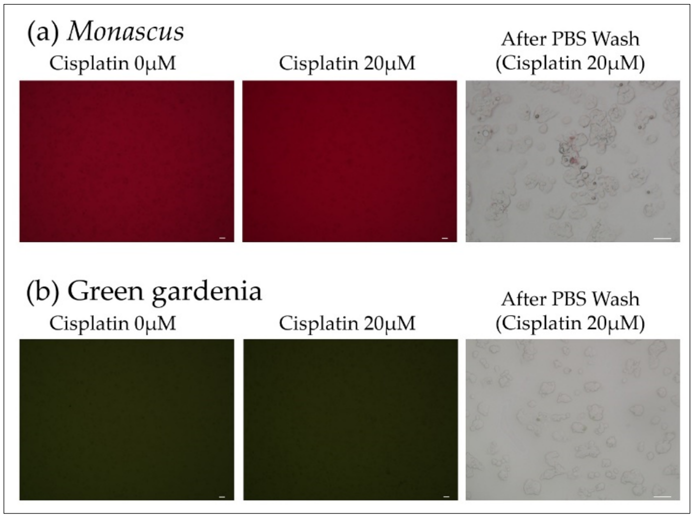

Figure S1. Confirmation of staining of dead cells (All scale bars designate 100 μm).
(a)*Monascus* (b)Green gardenia
Cells were observed with a bright-field microscope (4 × objective lens).

With *Monascus* pigment, staining of dead cells was clearly confirmed in the medium “After PBS wash”. Gardenia green showed staining of a very small number of dead cells but no clear staining.

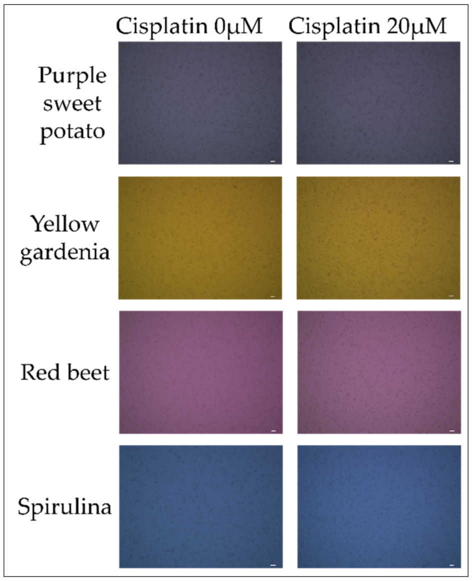

Figure S2. Confirmation of staining of dead cells (All scale bars designate 100 μm).
Cells were observed with a bright-field microscope (4 × objective lens).

No staining of cells was observed with any of the pigments with or without Cisplatin (Figure S2).

Based on the above, *Monascus* pigment was selected as a natural pigment that could be used as a reagent for viability assay in this study.

© 2020 by the authors. Submitted for possible open access publication under the terms and conditions of the Creative Commons Attribution (CC BY) license (http://creativecommons.org/licenses/by/4.0/).
